# Hip arthroscopy for femoroacetabular impingement in American football athletes: examining factors associated with outcomes at a mean follow-up time of 6.3 years

**DOI:** 10.1007/s00402-026-06551-6

**Published:** 2026-11-07

**Authors:** Andrew Atkinson, Michael Crick, Ryan Froom, Brody Westbrooks, Erin King, Eric Mussell, Anna Crawford, Campbell Aiken, Matthew Ithurburn, Michael Ryan, Benton Emblom

**Affiliations:** 1 American Sports Medicine Institute, Birmingham, United States; 2 Andrews Sports Medicine and Orthopaedic Center, Birmingham, United States; 3 School of Health Professions, University of Alabama at Birmingham, Birmingham, United States; 4 Prevea Health Orthopaedics and Sports Medicine, Green Bay, United States

**Keywords:** Femoroacetabular impingement, Hip arthroscopy, High school, Collegiate, American football, Patient-reported outcomes

## Abstract

**Introduction:**

Femoroacetabular impingement (FAIS) is a common cause of hip pain and performance limitation in athletes. Hip arthroscopy is the gold standard of treatment in professional American football athletes, with numerous studies demonstrating favorable outcomes and high return-to-sport (RTS) rates. However, limited outcomes data exists in collegiate athletes and fewer still in high school athletes. The purpose of this study was to evaluate long-term patient-reported outcomes (PROs), RTS rates, and predictors of outcomes following hip arthroscopy for symptomatic FAIS in high school and collegiate football athletes.

**Materials and methods:**

A retrospective cohort study was performed which included high-school and collegiate football athletes who underwent hip arthroscopy for symptomatic FAIS. Outcomes were assessed using the modified Harris Hip Score (mHHS), International Hip Outcome Tool-12 (IHOT-12), and Hip Outcomes Score-Sport Specific Subscale (HOS-Sport). Data regarding return to preinjury sport were also collected. Pre-operative radiographic data, including alpha angle, lateral center edge angle, and Tönnis grade, were collected.

**Results:**

This cohort had a mean post-operative follow up of 6.3 years, with mean PRO scores of 82.2 on the mHHS, 84.6 on the iHOT-12, and 88.3 on the HOS-Sport. PASS thresholds were achieved by 69%, 77% and 80% of athletes for the same outcomes, respectively. 60% of the cohort attempted to return to sport, and 91% of the athletes who attempted to return to preinjury level of sport were successful. Increased age at time of surgery and larger pre-operative alpha angles were associated with worse PRO scores at follow-up.

**Conclusions:**

Hip arthroscopy for FAIS in high school and collegiate football players yielded favorable long-term outcomes and RTS rates. This is similar to current outcomes data in professional athletes. Increased alpha angle and age at surgery were associated with worse outcomes, emphasizing the importance of early recognition and appropriate surgical treatment. Comparisons of outcomes between blocking and nonblocking positions may warrant further investigation in larger cohorts.

## Introduction

Femoroacetabular impingement syndrome (FAIS) is a hip joint pathology that is a common cause of pain and early joint degeneration in a variety of patient populations [4–6, 10]. FAIS is characterized by an abnormal morphology of osseous prominences on the femoral head-neck junction (cam type), acetabular rim (pincer type), or a combination of both (mixed type) [15, 41]. FAIS morphology can cause abnormal shearing forces to the articular cartilage and abnormal loading of the joint. This can result in degeneration of the hip joint, including delamination of the chondrolabral junction, articular cartilage damage, osteoarthritis, and labral tears [5, 6, 15]. A prior study found 60% of patients with acetabular deformities present with concomitant labral injuries [5]. Sports participation may play an important role in the development of cam lesions, with adolescent athletes demonstrating a higher prevalence of cam deformities than non-athlete adolescents [40, 43].

Current treatments for FAIS include both non-operative treatments for symptom control and operative interventions to repair or reconstruct the labrum and correct associated abnormal bony morphologies [4, 18, 47]. The current literature demonstrates surgical intervention may have superior clinical outcomes than non-operative treatment alone [3, 4, 9, 18, 47]. Additional research has concluded that treatment of labral deformities without addressing underlying bony deformities increases the likelihood of operative treatment of FAIS and increased need for revision [22]. Clinical outcomes of patients who underwent hip arthroscopy for FAIS-induced labral tears have been consistently favorable with high levels of patient satisfaction [36]. Hip arthroscopy has also been effective in athletic populations, demonstrating good clinical outcomes and high return-to-sport rates across various sports [1, 3, 25, 35, 37, 39]. For instance, Byrd and colleagues examined a cohort of 104 athletes (primarily football, basketball, soccer, and dance participants) and reported an average improvement of 25 points in the modified Harris Hip Score, rising from a score of 69 preoperatively to 94 postoperatively, and found that 87% of athletes returned to sport at their preinjury level [12].

In American football, the impact of untreated FAIS on athletes is well-documented, as intra-articular hip injuries such as labral tears can significantly affect performance and may be career altering [14, 46]. Feeley and colleagues found that injuries of the hip accounted for approximately 10% of all injuries in professional football, and intra-articular pathology resulted in a more significant loss of playing time than any other pathology of the hip in National Football League (NFL) players [14]. Related to outcomes, prior research has shown rates of successful return-to-sport (RTS) as high as 80–93% following hip arthroscopy in elite NFL athletes [31]. However, patient-reported and RTS outcomes of football athletes at the high school and collegiate level remains relatively unknown [14, 29, 33].

The primary objective of this study was to examine the clinical outcomes and RTS rates of high school and collegiate American football players after undergoing hip arthroscopy as a treatment for intra-articular hip pathology, including cam morphologies, pincer morphologies, and concomitant labral tears. Secondarily, we also examined potential factors associated with outcomes, including demographics, injury/surgery factors, and sport-related factors, such as football position. We hypothesized that, overall, athletes would demonstrate high rates of RTS that is similar to those of elite athletes, and that larger alpha angles and playing blocking positions would be associated with worse outcomes.

## Methods

### Patient population

Using Current Procedural Terminology (CPT) codes, we identified patients at Andrews Sports Medicine and Orthopaedic Center in Birmingham, Alabama who had been diagnosed with femoroacetabular impingement (FAIS) and treated with hip arthroscopy between 2013 and 2020. Inclusion criteria were as follows: 1) underwent hip arthroscopy at our institution, 2) between the ages of 14 to 35 years old at time of operative intervention, 3) participating in football competitively at the high school or collegiate level at the time of surgery, 4) skeletally-mature, 5) reached a minimum 2-year follow up, and 6) willing and able to provide informed consent. Patients with history of prior ipsilateral hip surgery, patients who underwent concomitant extra-articular procedures during their index procedure (including iliopsoas release, greater trochanteric bursectomy, or gluteus medius repair), and patients with advanced degenerative disease (defined by Tönnis grade >1) were excluded [11, 24]. Prior to starting this study, we obtained institutional review board approval (SterlingIRB; Project#7085), and all participants provided informed consent before the collection of their outcomes-related data.

### Surgical management and postoperative rehabilitation

All patients included in this study underwent hip arthroscopy performed by one of two fellowship-trained orthopedic surgeons whose clinical practice focused on pre-arthritic hip injuries and hip preservation (BAE and MKR). Both surgeons practiced at the same high-volume orthopaedic center. Patients included in this study underwent arthroscopic femoroplasty and/or acetabuloplasty for the treatment of femoroacetabular impingement (FAIS). Indications for this procedure included symptomatic pain and/or clicking and catching of the hip, alongside FAIS-related structural abnormalities (cam and pincer lesions) and/or labral tear. Additional procedures, such as labral repair, labral reconstruction, labral debridement, and/or subspinous decompression were performed as indicated. Labral repairs were performed using all-suture anchors, either knotted or knotless (2.0–3.0 mm SutureTak; Arthrex). Repair of the iliofemoral ligament (capsular closure) gained popularity during the surgical timeframe of included patients, and later patients underwent capsular closure more frequently. We obtained intraoperative data through electronic health record (EHR) review. All included patients were provided with one of two post-operative rehabilitation protocols based on the extent of their intraoperative findings. Patients with no labral involvement, labral debridement, or labral repair followed an accelerated rehabilitation protocol. In contrast, those who underwent labral reconstruction, ligamentum teres reconstruction, or capsular shift were provided a more conservative protocol which focused on addressing postoperative hip instability as this can result in a delayed return to full weightbearing. Clearance for full return to competition was granted when the patient could perform a single leg pick-up with a level pelvis, perform all sport-specific drills without symptoms, and had symmetric hip strength measured with handheld or isokinetic dynamometry.

### Demographics, injury characteristics, radiographic measurements, and subsequent operative procedures

We collected demographic, clinical, operative treatment, and sport-related data (age, BMI, acute/chronic injury, labral tear injury details, concomitant chondral injury, labral procedure and anchors, osteoplasty details, and football position at time of injury) for each eligible patient via electronic health record chart review. Prior to the index surgery, radiographs were also collected for each patient, including anteroposterior (AP), false profile, and Dunn views. AP views were available for every included patient, however some patients lacked Dunn and false profile views, due to evolving clinical assessment and preoperative planning. Whereas multiple radiographic measurements were used clinically for diagnostic and operative planning purposes (Tönnis/acetabular index, anterior wall index, head-neck offset, extrusion index, posterior wall index, anterior wall index, and neck-shaft angle), the following radiographic measurements were recorded/calculated from the AP view in all patients and reported in this study: AP alpha angle and lateral center edge angle [7, 19, 30, 44]. We utilized OrthoView software (Orthoview LLC, Jacksonville, Florida) to calculate the radiographic measurements. We also reviewed patients’ charts to determine if they had undergone a subsequent, ipsilateral hip procedure. If they had, they were considered to have met a primary endpoint in the study (i.e., need for revision procedure), and we purposefully did not obtain patient-reported outcomes (PROs) in these cases.

### Patient reported outcomes

Eligible patients who did not undergo subsequent ipsilateral hip procedures were contacted and asked to complete PROs. All PROs utilized have been previously validated to measure outcomes following hip arthroscopy for FAIS. The PROs administered were the International Hip Outcome Tool-12 (iHOT-12), the sports subscale of the Hip Outcome Score (HOS-Sport), and the Modified Harris Hip Score (mHHS) [16, 26, 42]. In addition, we calculated the proportions of athletes that met patient acceptable symptomatic state (PASS) thresholds for the mHHS (> 83.3), iHOT-12 (> 72.2), and HOS-Sport (> 76.4) reported in the literature [34]. In addition, we asked athletes a series of questions designed to assess return to sports and factors which contribute to return, including reasons for not returning, relevant contextual factors, and post-operative hip sport performance. We administered these measures via telephone calls or emailing a secure link and used Research Electronic Data Capture (REDCap) hosted at Ascension Health to collect and store all study-related data [20, 21]*.*

### Statistical analyses

All statistical analyses were performed using SPSS (version 29.0; SPSS Inc., Chicago, IL, USA). To determine meaningful missingness and better understand response bias, we compared demographics and clinical data between football athletes with and without completed follow-up data collection using independent t-tests for continuous variables or Chi-square tests for categorical/dichotomous variables. We summarized demographic, sport/position, injury, and treatment data for the included cohort with completed follow-up. Additionally, we summarized patient-reported outcomes data and calculated the proportions of football athletes that met PASS thresholds for the mHHS, iHOT-12, and HOS-Sport, as well as the proportions that returned to preinjury level of sport, and if not, reasons for inability to return [8, 48]. We compared patient-reported outcomes and proportions of return to preinjury sport between blocking and non-blocking position groups (blocking positions – offensive and defensive lineman and tight ends; non-blocking skill positions—quarterbacks, running backs, wide receivers, defensive backs, linebackers, safeties, fullbacks, and kickers) as well as between those that did and did not undergo capsule repair/closure during hip arthroscopy using independent t-tests and Chi-square tests, respectively. Additionally, we examined other potential predictors (age; follow-up time; pre-operative alpha angle; pre-operative lateral center edge angle) of outcomes using univariable linear regression (mHHS, iHOT-12, HOS-Sport scores) or logistic regression (return to preinjury sport). We assessed linearity and residual normality for linear models (residual and Q-Q plots) and linearity of the logit and goodness of fit for logistic models (Box-Tidwell and Hosmer–Lemeshow tests), and did not find meaningful violation of model assumptions. We considered groups differences or effects statistically significant when p-values were <0.05 for all analyses.

## Results

### Eligible patients and included cohort

**Fig. 1:**
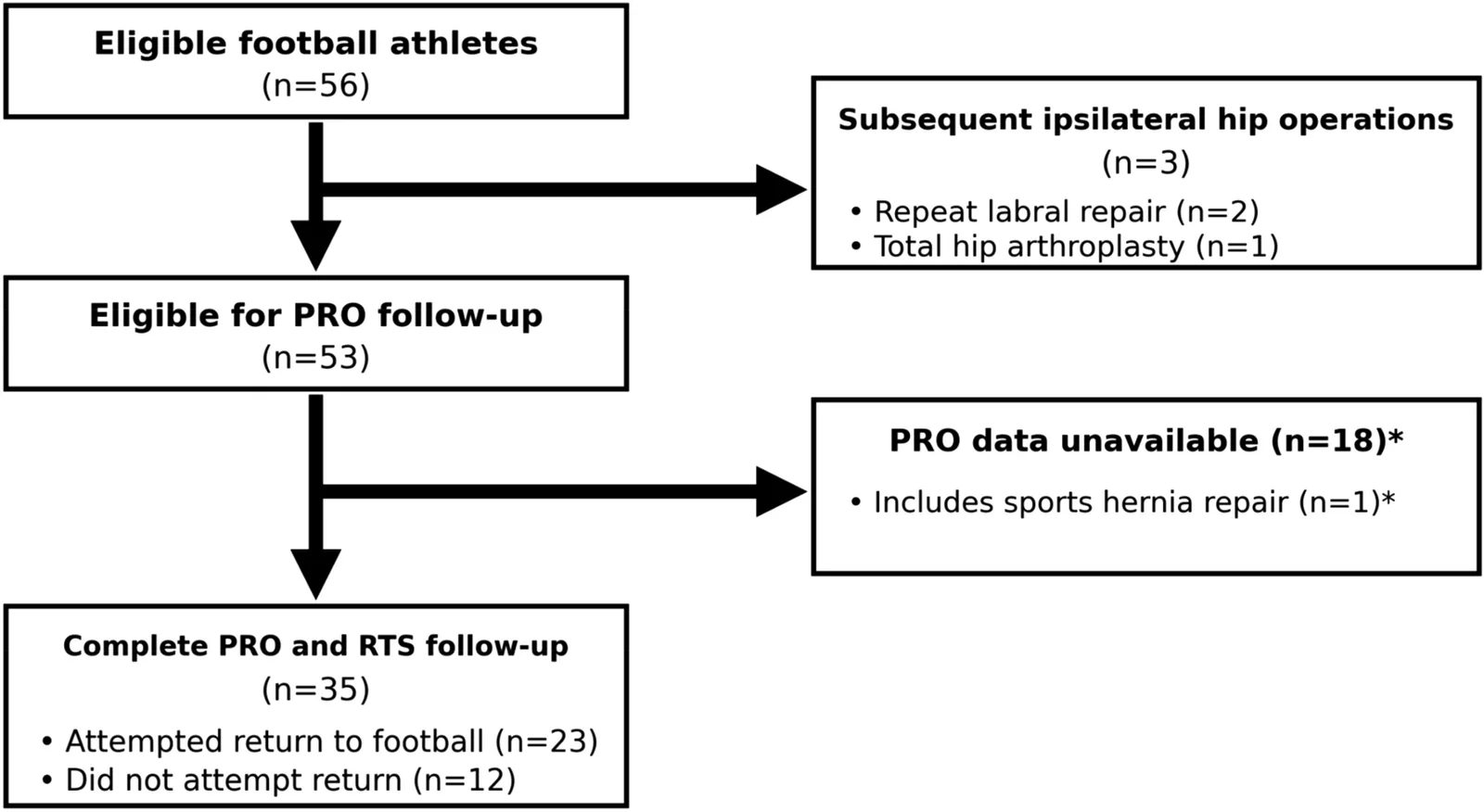
Flowchart of included football athletes. *CPT* current procedural terminology; *PRO* patient-reported outcome; *RTS* return to sport

A total of 56 football athletes underwent hip arthroscopy for FAIS, were a minimum 2-year post-operative at time of follow-up and met all other inclusion criteria. Three subsequently underwent ipsilateral hip procedures and were deemed to have met the study's endpoint. Of the remaining 53 football athletes, we collected PRO and RTS outcomes data for 35 with an average post-operative follow-up of 6.3 years. 18 of the athletes did not provide PRO data. This includes one athlete who underwent a sports hernia repair, which was not classified as a subsequent ipsilateral hip surgery as it is an extra-articular procedure. Including those with PRO data collection (n = 35) and those with subsequent procedures (n=3), we were able to follow up with a total of 68% of eligible football athletes (38/56; Fig. 1).

**Table 1:** Demographic and clinical data for all included football athletes

Variable	Included Football Athletes (n = 35)
Sex, male	35 (100%)
Age at surgery, years	18.4 ± 2.9
Follow-up time, years	6.3 ± 2.4
Level of competition High school Collegiate	27 (77%) 8 (23%)
Position Linebacker Offensive lineman Safety Defensive back Quarterback Kicker Other	9 (25%) 6 (17%) 4 (11%) 3 (9%) 3 (9%) 3 (9%) 7 (20%)
Femoral head chondral defect present	1 (3%)
Acetabular chondral defect present	6 (17%)
Alpha angle, degrees	59.3 ± 16.3
Lateral center edge angle, degrees	26.3 ± 5.3
Type of osteoplasty performed	*Acetabuloplasty only*: 4 (11%) *Femoroplasty only*: 18 (52%) *Both*: 13 (37%)
Labral procedure performed	*Repair*: 33 (94%) *Debridement only:* 1 (3%) *Reconstruction*: 1 (3%)
Extra-articular impingement procedure performed	*Subspine decompression only*: 1 (3%)
Capsule closure performed	22 (63%)

Data are presented as n (%) or mean ± standard deviation

Football athletes with PRO data collection (n = 35) did not differ from those without PRO data collection (n = 18) in age at surgery (p = 0.10), time since hip arthroscopy (p = 0.75), preoperative alpha angle (p = 0.48), nor preoperative center edge angle (p = 0.41). Additionally, when comparing athletes with and without PRO data collection, there were no differences in the proportions of femoral head chondral defects (p = 0.09), acetabular chondral defects (p = 0.33), capsule closure/repair (p = 0.42), nor the distribution of osteoplasty type (p = 0.96). There were three total revision procedures- 2 labral repairs and 1 total hip arthroplasty. One of these three patients was able to return to preinjury level of sport between the primary and revision procedures. The other two did not attempt to return to preinjury sport. Both patients who underwent revision labral repairs underwent surgery at an outside clinic.

### Demographic and clinical/surgical data

Demographic, sport/position, clinical, radiographic, and surgical treatment data for the included cohort of football athletes with PRO data (n = 35) are shown in Table 1. All included football athletes were male, with an average age at surgery of 18.4 years. Approximately three-quarters of football athletes competed at the high school and the remaining one-quarter competed at the collegiate level. Regarding football positions, the most common positions were linebacker, offensive lineman, and safety. During hip arthroscopy, the majority of athletes underwent isolated femoroplasty as a part of their FAIS-related surgery (52%), nearly all (94%) underwent labral repairs, and hip capsule closure was performed in 63% of included athletes.

### Patient-reported and return-to-sport outcomes

Mean patient-reported outcome scores (mHHS; iHOT-12; HOS-Sport) as well as the proportions of football athletes meeting PASS thresholds and RTS outcomes are shown in Table 2. In total, 21 of 35 football athletes returned to preinjury level of participation in football (21/35; 60%). However, twelve football athletes did not attempt to return to preinjury football participation following their hip arthroscopy. The most common reasons for not attempting to return included graduation and not being talented enough to move to the next level (10 of 14), followed by decreased interest or other personal reasons (2 of 14; Table 2). Of those who attempted to return, 21 of 23 (91%) were able to return to preinjury level of participation. Two athletes attempted to return but were unable to, citing hip pain and/or decreased hip-related performance in sport following surgery.

**Table 2:** Patient-reported and return-to-sport outcomes

Variable	Included Football Athletes (n = 35)
mHHS Score at follow-up* Proportion meeting PASS cutoff	82.2 ± 13.7 69%
iHOT-12 score at follow-up Score at follow-up* Proportion meeting PASS cutoff	84.6 ± 23.8 77%
HOS-Sport score at follow-up Score at follow-up* Proportion meeting PASS cutoff	88.3 ± 19.0 80%
Proportion of athletes able to return to preinjury sport who attempted to return following surgery^#^	91% (n = 21/23)
Reason for unsuccessful RTS (n = 14^) Graduated but not talented enough for next level Limited by hip/surgery Personal reasons	10/14 2/14 2/14

^*^Data are presented as mean ± standard deviation; ^#^Number of athletes that attempted to return-to-sport following hip arthroscopy excludes those that did not attempt to return due to graduation and insufficient talent to proceed to the next level or for personal reasons, such as lost interest (n = 12); ^n=14 did not return to preinjury sport for all reasons

### Group comparisons and predictors of outcomes

Athletes in blocking positions reported lower scores across all questionnaires at follow-up (mHHS, iHOT-12, HOS-Sport) compared to non-blocking positions, however these group differences were not statistically-significant (Table 3). Likewise, there were no statistical differences in the proportions meeting PASS cutoffs for the mHHS (blocking: 63%; non-blocking: 70%; p = 0.67), iHOT-12 (blocking: 75%; non-blocking: 78%; p = 0.87), nor HOS-Sport (blocking: 63%; non-blocking: 85%; p = 0.16) between blocking and non-blocking position football athletes. Lastly, there were no statistical differences in the proportions that returned to preinjury level of participation in football between those in blocking and non-blocking positions (p = 0.87).

**Table 3:** Patient-reported outcomes data comparisons between football position groups

	Blocking positions (n = 8)	Nonblocking positions (n = 27)	*p*^#^
mHHS	77.0 ± 21.3	83.7 ± 10.7	0.23
IHOT-12	75.8 ± 34.4	87.2 ± 19.7	0.24
HOS-Sport	80.1 ± 26.5	90.7 ± 16.0	0.17

Data are presented as mean ± standard deviation; mHHS, modified Harris Hip Score; iHOT-12, International Hip Outcome Tool-12; HOS-Sport, sports subscale of the Hip Outcome Score

Related to capsule repair/closure, there were no statistical differences in all questionnaires at follow-up (Table 4). Additionally, there were no statistical differences in the proportions meeting PASS cutoffs for the mHHS (repair: 68%; no repair: 67%; p = 0.93), iHOT-12 (repair: 73%; no repair: 83%; p = 0.49), nor HOS-Sport (repair: 77%; no repair: 83%; p = 0.68) between those that underwent and did not undergo capsule repair/closure. Lastly, there were no statistical differences in the proportions that returned to preinjury level of participation in football between those that underwent and did not undergo capsule repair/closure (p = 0.97).

**Table 4:** Patient-Reported Outcomes Data Comparisons Between Those With and Without Capsule Repair

	Capsule Repair (n = 22)	No Capsule Repair (n = 13)	*p*^#^
mHHS	82.5 ± 12.3	80.9 ± 16.9	0.76
IHOT-12	84.0 ± 23.0	84.4 ± 26.7	0.97
HOS-Sport	88.9 ± 15.6	85.9 ± 25.2	0.67

Data are presented as mean ± standard deviation; mHHS, modified Harris Hip Score; iHOT-12, International Hip Outcome Tool-12; HOS-Sport, sports subscale of the Hip Outcome Score

Examining potential predictors of outcomes, increased age at surgery was associated with lower/worse iHOT-12 scores at follow-up (B=− 2.8 [− 5.5, − 0.09]. R^2^ = 0.12, p = 0.04) and trended toward being associated with lower/worse mHHS scores at follow-up (B = − 1.4 [− 3.0, 0.23]. R^2^ = 0.09, p = 0.09). Larger pre-operative alpha angles were associated with worse mHHS scores (B=− 0.29 [− 0.57, − 0.01]. R^2^ = 0.11, p = 0.04), worse iHOT-12 scores (B = − 0.54 [− 1.03, − 0.04]. R^2^ = 0.13, p = 0.03), and worse HOS-Sport scores (B = − 0.48 [− 0.87, − 0.09]. R^2^ = 0.17, p = 0.02) at follow-up. Lastly, older age at surgery was also associated with lower odds of returning to preinjury level of sport following surgery (odds ratio = 0.61 [0.41,0.91, p=0.02). There were no significant associations between follow-up time or pre-operative lateral center edge angles and any questionnaire score at follow-up. Additionally, follow-up time, pre-operative alpha angles, and pre-operative center edge angles were not associated with the ability to successfully return to preinjury level of sport.

## Discussion

This retrospective cohort study provides insight into clinical outcomes and RTS rates in American high school and collegiate football players following hip arthroscopy for treatment of FAIS and associated labral tears. Hip arthroscopy has demonstrated significant improvements in clinical outcomes in athletic populations [49]. The cohort in this study had a mean age of approximately 18 years old and consisted exclusively of male football players, with three quarters at the high school level and the remainder at the collegiate level. These patients demonstrated high rates of return to preinjury level of sport (amongst those that attempted) and favorable long-term outcomes over a follow-up time of more than 6 years. The cohort’s mean PRO scores were 82.2 for the mHHS, 84.6 for the iHOT-12, and 88.3 for the HOS-Sport, and met PASS thresholds at a rate of 69%, 77%, and 80% for each measure, respectively. Most of the patients underwent labral repair (95%), over half had an isolated femoroplasty (53%), and nearly two thirds had a capsular closure. In total, these results are favorable for hip arthroscopy as a treatment for symptomatic FAIS in this cohort.

Outcomes of hip arthroscopy to treat FAIS have been well-documented in professional football athletes, with high RTS rates between 90 and 93% [28, 31]. For example, Nwachukwu and colleagues evaluated 40 NFL players that underwent hip arthroscopy for FAIS and reported a RTS rate of 93% for this case series [29]. Similar data is seen in professional athletes of other sports. Elwood and colleagues reported a RTS rate of approximately 94% in their systematic review of nearly 1,000 professional athletes following hip arthroscopy [13]. The results of the present study parallel the results previously described in other populations of athletes, as we found that 91% of high school and collegiate football athletes who attempted to return to preinjury level of sport were successful. However, of the 14 athletes who did not attempt to return to preinjury sport/preinjury level of sport, we found that 10 football athletes did not attempt to return to their preinjury level due to graduation and/or insufficient talent to move to the next level of competition (e.g., from high school to collegiate or collegiate to professional football). These findings are particularly encouraging given the lower level of competition for our football cohort, which included likely limited access to comprehensive rehabilitation, physical therapy, and relevant resources compared to professional athletes. Additionally, PRO scores at final follow-up in our cohort were similar to the current literature for professional athletes that reported average values on the mHHS and HOS-Sport of 92 and 82 respectively [13]. Importantly, this cohort did not have baseline/pre-operative data, and we were unable to calculate minimal clinically important difference (MCID) values, limiting the ability to quantify improvement from before to after hip arthroscopy**.** Overall, these data suggest that hip arthroscopy may be similarly effective as a treatment option for symptomatic FAIS in high school and collegiate football athletes as it is in professionals.

Examining predictors of outcomes, age and preoperative alpha angle were associated with PRO scores at follow-up. Specifically, older age was associated with significantly lower IHOT-12 scores and trended toward being associated with lower mHHS scores. Older age was also associated with lower RTS rates. One factor which may contribute to decreased RTS rates in older athletes is the limited number of athletes who continue to play at the next competitive level unable to transition from either high school to college football or college to professional football. Additionally, larger preoperative alpha angles were associated with significantly lower scores on all three PROs at follow-up. A prior biomechanical study showed that larger cam deformities caused increased tensile strain and peak shear stress on the acetabular cartilage [45]. Our findings are also in line with the current literature related to alpha angles and outcomes data. Martins and colleagues’ systematic review found that increased alpha angles were associated with more severe hip chondral damage [27]. Regarding association with PROs, Gürsan and colleagues demonstrated that higher postoperative alpha angles were associated with worse outcomes scores, with patients who had an alpha angle of <48.3 ⁰ reached the mHHS PASS threshold at a significantly greater value than those with an alpha angle of >48.3 ⁰ (p = 0.002) [17]. Overall, these studies are consistent with our findings of worse outcomes associated with larger preoperative alpha angles.

In our study, capsular closure and lateral center edge angle (LCEA) had no significant association with outcomes. The hip capsule is a complex framework of multiple ligaments that serve to stabilize the hip joint [32, 50]. The current evidence is mixed regarding whether closure of the hip capsule is associated with better postoperative outcomes [12]. A meta-analysis of more than 5,000 patients reported better clinical outcomes with capsular closure [38]. Specifically, the proportion of patients with successful outcomes, which was determined by a combination of satisfaction scores and PROs, was 83% in those without closure and 90% in those with closure [38]. In contrast, Atzmon and colleagues found no difference in satisfaction or PROs between those who underwent capsular closure and those who did not [2]. Regarding LCEA, this radiographic measurement is used to determine acetabular coverage, with measurements <20⁰ indicating under-coverage and associated with hip dysplasia, while measurements >39⁰ are considered over-coverage and associated with pincer deformities [23]. Relating to outcomes, Çeltik and colleagues demonstrated that patients with preoperative LCEA ≤ 24.8 ⁰ had higher rates of achieving the mHHS PASS threshold than those with LCEA > 24.8 ⁰ (p=0.01) [51]. In our cohort, there was no significant association between LCEA and outcomes. However, our cohort was a relatively small cohort of entirely male athletes, with LCEA in a mostly “normal” range.

We originally set out to examine the impact of football position, dividing the cohort into two subgroups: blocking and nonblocking athletes. Blocking positions included offensive lineman, defensive lineman, and tight ends, whereas nonblocking positions included defensive back, kicker, linebacker, quarterback, running back, safety, and wide receiver. We hypothesized that position-specific biomechanical demands in American football may create predispositions for distinct hip injury patterns. We also speculated that these position-specific biomechanics might have influenced not only injury mechanisms but also responses to surgical treatment, rehabilitation, and outcomes overall. Despite moderately large differences in group means, our position group comparisons were underpowered, based on a small number of blocking position athletes that underwent hip arthroscopy for FAIS. We plan for future studies with a greater number of participants to examine the role of football position on injury characteristics and treatment outcomes.

### Limitations

This study has several limitations that are important to recognize. First, an important limitation is that baseline questionnaire data were not collected for this cohort, which limited our ability to determine pre- to post-surgical improvements, as well as the proportions meeting clinically-relevant thresholds such as minimal clinically important difference (MCID). This limited our ability to quantify how effective surgical treatment was in relation to pre-operative function. We used PASS rates as an alternative method to measure outcomes; however, we recognize that they should not be used as a substitute for a validated measure of improvement or treatment effect, such as MCID. Secondly, the smaller sample size resulted in limited statistical power, especially for the positional group analysis. Because of our small sample size, we did not adjust for multiple comparisons in our regression analyses, due to concern of missing meaningful associations and balancing Type 1 and Type 2 error. Because of this, the association results should be considered with caution. Thirdly, as this is a retrospective case series with long-term outcomes data, there is the possibility of recall bias. This cohort was also heterogeneous, as it included football players at varying levels (high school and college) and position, capsular management and osteoplasty techniques. Next, preoperative alpha angles were measured using AP pelvis radiographs because Dunn views were not available for every athlete. Alpha angle measurements depend on the radiographic projection used, and AP views may not capture the greatest cam deformity. This limits the comparison that can be made with studies who use Dunn or other lateral views and should be considered when interpreting the association between preoperative alpha angle and follow-up outcomes. Lastly, we did not have access to or collect post-operative objective data such as physical exam attributes and imaging, which limited our conclusions regarding outcomes to just those reported by the athletes. Despite these limitations, this study provides long-term outcomes data in a previously understudied population of high school and collegiate football athletes.

## Conclusions

The findings of this study demonstrated positive outcomes following hip arthroscopy for the treatment of FAIS and labral pathology in high school and collegiate American football players that are similar to those observed in professional athletes. We also found that increased alpha angle and age at surgery were associated with worse PROs. Future research should investigate position-specific differences in outcomes in larger cohorts, as well as examining changes in preoperative and postoperative PROs to better quantify surgical benefit in football players.

## Data Availability

No datasets were generated or analysed during the current study.
